# Rare origin of the obturator artery from the external iliac artery with two obturator veins

**DOI:** 10.1590/1677-5449.005116

**Published:** 2016

**Authors:** Kiyoshi Goke, Lucas Alves Sarmento Pires, Tulio Fabiano de Oliveira Leite, Carlos Alberto Araujo Chagas

**Affiliations:** 1 Universidade Estácio de Sá – UNESA, Departamento de Anatomia, Rio de Janeiro, RJ, Brazil.; 2 Universidade Federal Fluminense – UFF, Departamento de Morfologia, Niterói, RJ, Brazil.; 3 Universidade de São Paulo – USP, Departamento de Radiologia, São Paulo, SP, Brazil.

**Keywords:** anatomical variation, obturator artery, obturator vein, artéria obturatória, veia obturatória, variação anatômica

## Abstract

The obturator artery is a branch of the internal iliac artery, although there are reports documenting variations, with origin from neighboring vessels such as the common iliac and external iliac arteries or from any branch of the internal iliac artery. It normally runs anteroinferiorly along the lateral wall of the pelvis to the upper part of the obturator foramen where it exits the pelvis by passing through said foramen. Along its course, the artery is accompanied by the obturator nerve and one obturator vein. It supplies the muscles of the medial compartment of the thigh and anastomoses with branches of the femoral artery on the hip joint. We report a rare arterial variation in a Brazilian cadaver in which the obturator artery arose from the external iliac artery, passing beyond the external iliac vein toward the obturator foramen, and was accompanied by two obturator veins with distinct paths. We also discuss its clinical significance.

## Resumo

A artéria obturatória é um ramo da artéria ilíaca interna, embora haja grande variabilidade a respeito de sua origem, pois tal vaso pode surgir de diversas artérias vizinhas, como a artéria ilíaca comum e a artéria ilíaca externa, assim de como qualquer ramo da artéria ilíaca interna. Normalmente, a artéria obturatória corre anteroinferiormente pela parede lateral da pelve até a porção superior do forame obturatório, ponto em que sai da pelve. No seu trajeto, a artéria é acompanhada pelo nervo e veia obturatórios. Ela supre os músculos do compartimento medial da coxa e possui anastomoses com ramos da artéria femoral na articulação do quadril e coxa. Este trabalho visa relatar e discutir os aspectos clínicos de uma variação incomum na artéria obturatória, na qual ela se origina da artéria ilíaca externa acompanhada de duas veias obturatórias com trajetos distintos.

## INTRODUCTION

The obturator artery (OA) is usually a branch of the anterior division of the internal iliac artery (IIA), running medially to the lateral wall of the pelvis to reach the obturator canal. This artery emits iliac branches (to the iliac fossa), a vesicle branch (to the urinary bladder), and a pubic branch, which anastomoses with the inferior epigastric artery (IEA) and the contralateral pubic branch. As it exits the pelvis, the OA divides into anterior and posterior branches.^1^ The ureter and the vas deferens cross the artery medially and the vessel is followed by the obturator nerve (ON) and the obturator vein (OV).^1^

The OV is formed in the proximal portion of the adductor region, and runs to the pelvis through the obturator foramen (OBF) in the obturator canal before running posteriorly and superiorly on the lateral wall of the pelvis, inferior to the OA, and crossing the ureter and the IIA to join the internal iliac vein (IIV).^1^^,^ 2 The OA is usually inferior to the ON and superior to the OV, although they do not run in parallel with each another.^3^

These vessels are often involved in anatomic variations affecting their origins and paths and so careful studies are needed to ensure success before vascular and orthopedic procedures. It is also crucial to study these vessels in view of the many reports in the literature describing pelvic fractures associated with hemorrhage and iatrogenic lesions, with special regard to the “corona mortis” arterial variation.^1^^,^ 2 ^,^ 4 ^-^ 9

The aim of this study is to present a case of arterial variation in which the OA arose from the external iliac artery (EIA), passing beyond the external iliac vein toward the OBF, and was accompanied by two OVs with distinct paths.

## CASE REPORT

**Figure 1:**
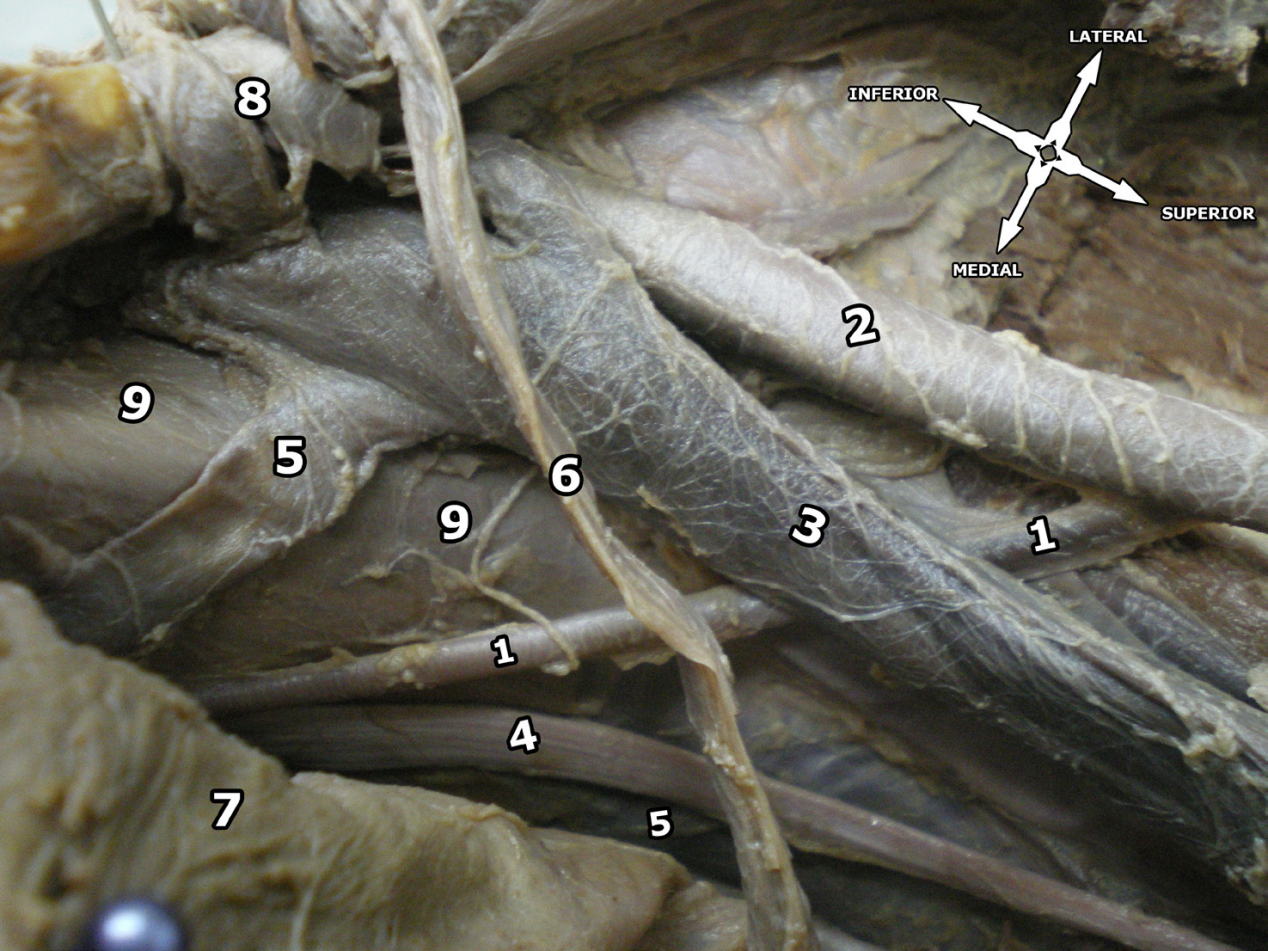
Superior view of the right pelvic region. 1 = obturator artery; 2 = external iliac artery; 3 = external iliac vein; 4 = obturator nerve; 5 = obturator veins; 6 = vas deferens; 7 = urinary bladder; 8 = inferior epigastric artery and vein; 9 = psoas major muscle.

During dissection of the right pelvic region of a male cadaver fixed with a 10% formalin solution, we observed that the OA originated from the EIA and passed laterally to the EIV, before crossing the pelvic brim and descending anteriorly into the OBF (Figure 1). Medially, the OA was related to the right ureter and right vas deferens. On exiting the OBF, it divided into anterior and posterior branches. None of the remaining branches exhibited any variations. The total length of the right OA was 9.8 cm. It was given off at a distance of 6 cm from the point of bifurcation of the right common iliac artery.

**Figure 2:**
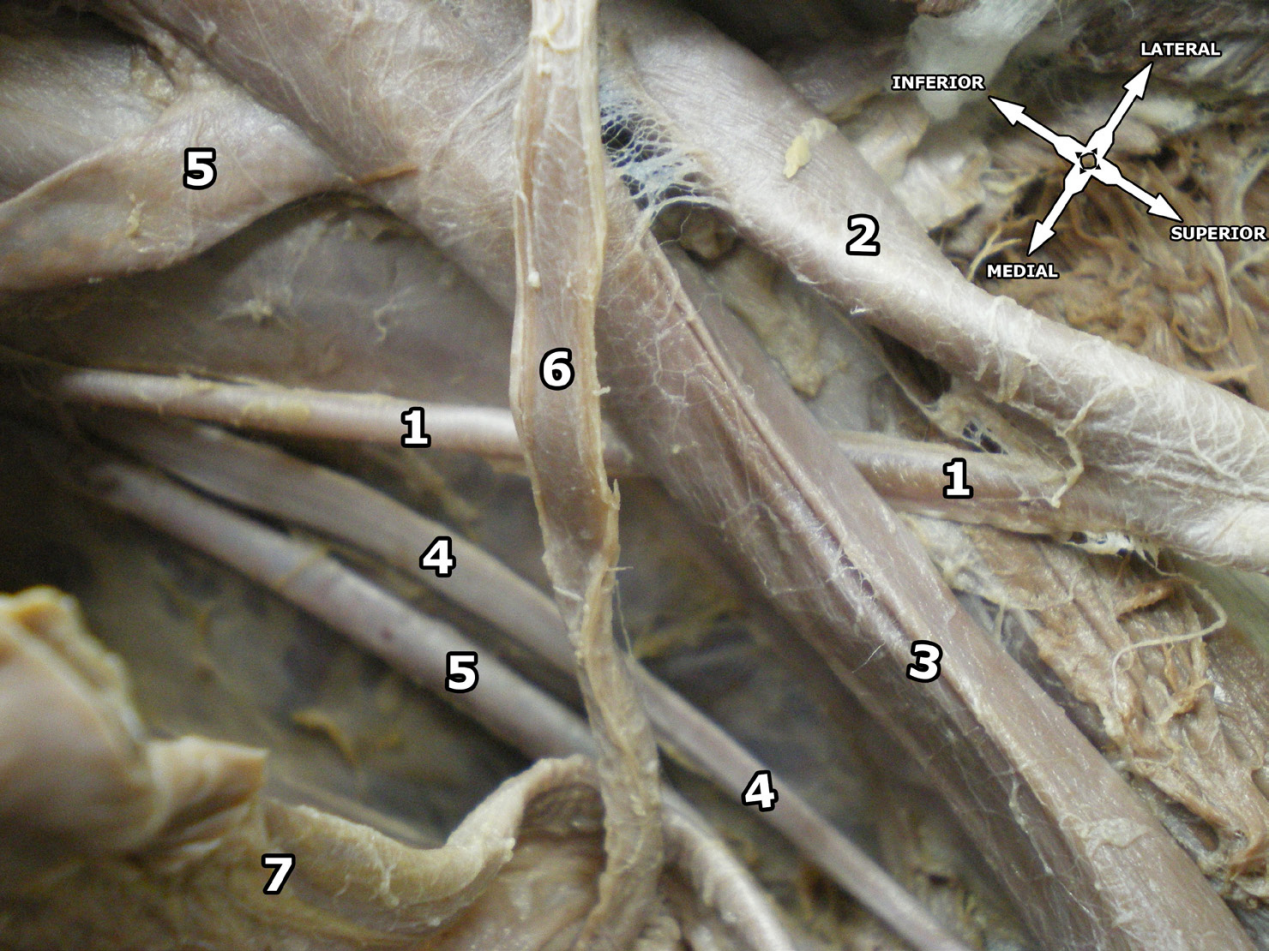
Superior view of the right pelvic region. 1 = obturator artery; 2 = external iliac artery; 3 = external iliac vein; 4 = obturator nerve; 5 = obturator veins; 6 = vas deferens; 7 = urinary bladder.

The origins of the IEA and the deep circumflex iliac artery were as normal. The ON entered the OBF below the artery (Figure 1). There were two OVs. One terminated into an anterior branch of the IIV, while the other one terminated in the EIV (Figure 2).

## DISCUSSION

During embryonic life some arterial channels appear (or enlarge) and disappear (or retract) and the result of this process will dictate the vascular territory of each artery.^10^^,^ 11 The dorsal root of the umbilical artery gives rise to two arterial plexuses (the abdominal and the pelvic). The pelvic plexus gives origin to the EIA and the IIA, and it appears that the OA is formed as a result of uneven growth of an anastomosis between these vessels.^7^^,^ 12 Thus, persistence of the arterial channels, combined with the fact that some authors believe that the OA arises later in the embryo, could be the answer to the question of why the OA can have so many different origins and trajectories.^11^^,^ 12

According to the literature, the OA can arise from the common iliac artery, EIA, IEA, inferior gluteal artery, internal pudendal artery (or a common trunk between both arteries), iliolumbar artery, or even from the superior gluteal artery.^4^^,^ 7 It can also have two or even three origins, and one can also find an accessory OA.^4^^,^ 7 Furthermore, Bergman et al.^4^ state that this artery can also have different origins on the left and right sides in the same person.

Sañudo et al.^7^ performed a meta-analysis in which they addressed all reported variations of the OA in the literature and created a classification consisting of 6 different types. These types are shown in Table 1. According to Sañudo et al., types A and B are the most common (35.5 and 22.5%, respectively).^7^ According to this classification, our variation is Type E, the second rarest type described in the literature (1.7% of cases).^7^ Type F, in which the OA rises from the femoral artery, has only been reported once^13^, thus, further research into these vessels is clearly important for vascular surgeons.

**Table 1:** Variability pattern of the Obturator artery as proposed by Sañudo et al.^7^

**Type**	**Description**
Type A)	The OA arises from the anterior division of the IIA (most common).
Type B)	The OA arises from the IEA.
Type C)	The OA is a branch of the posterior division of the IIA.
Type D)	The OA arises from the IIA, above its final branching.
Type E)	The OA arises from the EIA.
Type F)	The OA arises from the femoral artery (least common).

OA: obturator artery; IIA: internal iliac artery; IEA: inferior epigastric artery; EIA: external iliac artery.

The OV can sometimes connect with the inferior epigastric vein or the femoral vein^4^ and, according to Gilroy et al., the OV is often subject to variations and in the majority of their sample the cadaveric pelvis they dissected had one “normal” OV and one “aberrant” OV.^5^ Knowledge of the variability of the OV is important because of the possibility of iatrogenic injuries during catheterisation.^2^

The variable origins of the OA can also influence its relationship with the ON and OV. For instance, when it arises from the EIA, the artery would be placed superiorly in relation to such structures, which would affect the surgeon during delicate procedures.^7^

The corona mortis variation is a vascular connection between the obturator and the inferior epigastric vessels (or directly to the EIA) in which either the artery or vein (and sometimes both) forms an anastomosis near the superior pubic ramus. It is also a connection between the EIA and the OA.^14^ Its clinical significance is crucial, due to the fact that it can be damaged during pubic fractures or surgery.^15^ Furthermore, gynecologic and urologic surgeries, extraperitoneal inguinal hernioplasty, laparoscopic herniorrhaphy, Burch procedures, arterial embolization, periacetabular osteotomies, traumas, and resection of tumors require extensive knowledge of the pelvic vascular anatomy because of the risks of bleeding and damage to other structures, such as the ON, and since the pelvic wall vessels are often obscured by preperitoneal tissue, fat, and lymphatics, it is critical that surgeons anticipate these variations.^5^^-^ 9 ^,^ 16

Obturator bypass is a procedure that was initially described with the premise of treating mycotic aneurysms, but has since become popular and has been widely used to treat any form of injury to the femoral and iliac systems.^17^ In our case, the OA arose from the EIA, which means that there is no direct connection between the external iliac and internal iliac systems through the OA. Nevertheless, this anatomical variation could be a source of collateral circulation to the lower limb in patients with chronic obstruction of the external iliac distally or of the common femoral arteries, as the OA would function as a natural anatomical bypass.

The OA variant reported here is very important surgically because it could cause dangerous complications during femoral ring procedures or laparoscopic interventions, due to its unusual trajectory and the presence of a supernumerary OV.
